# Sensitivity and Expected Change of Commonly Used Social Communication Measures in Longitudinal Research of Young Autistic Children

**DOI:** 10.1007/s10803-025-06863-3

**Published:** 2025-05-10

**Authors:** Kyle Sterrett, Maria Pizzano

**Affiliations:** 1 Semel Institute for Neuroscience and Human Behavior, University of California, Los Angeles (UCLA), 760 Westwood Plaza, Los Angeles, CA 90024 USA; 2 University of Minnesota, Masonic Institute for the Developing Brain, 2025 E River Pkwy, Minneapolis, MN 5541 USA

**Keywords:** Autism spectrum disorder, Longitudinal, Psychometrics, Social-communication, Meta-analysis

## Abstract

Social communication measures used to track change in autistic children have not undergone rigorous psychometric evaluation. There is little data on their expected change or sensitivity to change. Meta-analytic techniques were used to examine sensitivity to change and expected change over time and whether these are influenced by factors like children’s age and the presence of intervention. Pooled effect sizes were generated within measures, rather than within broader constructs. Change over time was small to medium, although there was variability. Most outcomes were not sensitive to change over time. Change in some measures was influenced by child characteristics and methodological characteristics of included studies such as study quality and the method of scoring measures (e.g., using age-equivalents versus standard scores). Tests measuring similar constructs can vary in their expected change, and so care is needed when selecting them.

The study of psychometrics remains central to the rigor of the science of psychology. One of the most consistent critiques over the last 20 years of autism research has been the lack of standardized, autism-specific measures that are sensitive to change, reliable, and valid (Grzadzinski et al., 2020). In a recent review, 131 different outcome measures (excluding observational and study-specific measures) were identified (McConachie et al., 2015). Only twelve measures across all outcome types (e.g., adaptive functioning, autism behaviors, language, cognitive ability) were found to have adequate evidence of validity to measure their intended constructs (Mokkink et al., 2010; McConachie et al., 2015). Despite evidence of validity in that small subset of measures, McConachie and colleagues (2015) concluded that there is no evidence that they are sensitive to change over short periods of time (e.g., 3 months), a typical length of an early intervention trial for young autistic children. The use of measurement tools that have strong psychometric properties, such as sensitivity to change over time, is therefore needed to better understand the trajectories and behavioral intervention outcomes of autistic children.

Social-communication measures represent the most common outcomes in early intervention trials (Sandbank et al., 2020), yet high quality and validated, social communication measures are as sparse as those representing other constructs. This speaks to the need for more rigorous psychometric evaluations of social-communication measures to better understand their use as outcomes. For the purposes of this study, we are defining social communication more broadly than the diagnostic criteria for autism to include measures of expressive and receptive language. There have been recent attempts to develop psychometrically-validated measures of social-communication change such as the Eliciting Language Samples for Analysis (ELSA; Barokova et al., 2021) to measure spoken-language progress and the Autism Impact Measure (AIM; Mazurek et al., 2020) and the Brief Observation of Social Communication Change (BOSCC; Grzadzinski et al., 2016; Kim et al., 2019) to measure changes in autism related behaviors. The development of these measures represents a positive step forward. However, there are reasons to expect issues surrounding outcome measurement to persist. First, there is a lack of consensus as to what appropriate outcomes should be (Tager-Flusberg & Kasari, 2013). It is unlikely that there will be full, consistent, and timely adoption of any single measure purported to assess any particular construct across the research community. Institutional memory, strong opinions about the appropriateness of specific measures, and the need for consistency and comparability across past studies often drives the selection of outcomes and will continue to lead to the use of the wide array of measures.

## Need for Social Communication Measures Sensitive to Change

To supplement ongoing measure-development efforts, it is important to continue systematic efforts to better understand, evaluate, and provide data about the performance of currently available assessments used to measure change in social-communication skills. However, to date, there have been no attempts to systematically measure the sensitivity to change over time or the expected change over time of commonly used measures of social communication. Here, sensitivity to change is operationalized as whether the amount of change on an outcome is related to the length of the measurement period—whether more progress is made in longer studies. Expected change is defined as the average effect over a measurement period, independent of its length. Having a benchmark for expected change is important because it establishes a metric to compare the results of new trials and helps place the results of previous trials in a more appropriate context. No such benchmark exists in the early intervention literature for autistic children.

Considerable evidence exists showing that change over time in children’s social communication is affected both by the individual characteristics of the autistic participants as well as broader contextual factors related to the study itself. For example, individual factors such as children’s age differentially affect the amount of change over time observed for children enrolled in treatment trials, with younger children changing more over time (Guthrie et al., 2023). Contextual factors, such as the treatment provider, also affect the amount of observed change. Children who are enrolled in university-based treatments tend to make more change over time compared to community-based treatments (Nahmias et al., 2019). However, little to no difference was seen between university-based and community-based treatments in a recent large-scale randomized-controlled trial (Rogers et al., 2019). Community-based treatments have improved considerably in recent years and further investigation of this relationship is needed. The methodological quality of the studies measuring specific constructs also influences change over time (Sandbank et al., 2020). Lastly, there has been increased attention given to the influence of the way measures are scored and their change over time, warranting further consideration and evaluation of the use of norm-referenced measures to track change over time (Farmer et al., 2020). It is therefore important to understand how participant characteristics (e.g., chronological age) and study-level characteristics (e.g., enrollment in treatment) influence the magnitude of change and sensitivity to change over time of outcome measures.

To address these gaps, a systematic review and meta-analysis was conducted. The primary aim of the study was to determine the average expected change over time (effect size) of individual measures of social-communication change in longitudinal research and intervention trials. Meta-analyses commonly average effect sizes of the change over time of specific constructs such as non-verbal IQ or language that are represented by multiple different measures (Eldevik et al., 2009; Virués-Ortega, 2010; Sandbank et al., 2020). However, there is no evidence that different measures should be considered psychometrically comparable. The present data are organized to evaluate change over time within specific measures rather than across broader constructs. Secondary aims evaluated whether other factors -- the way measures are scored (i.e., standardized, age equivalent, raw or developmental quotient), type of intervention, age, and study quality -- are related to change in social-communication measures.

## Method

### Search Strategy

A systematic search of three large databases (PsychINFO, Web of Science and PubMed) was conducted to cull relevant articles. The full list of terms used for the search is included in Appendix A. The first layer of search terms consisted of diagnostic labels related to autism (e.g., autism, autistic, ASD, Pervasive Developmental Disorder (PDD), PDD), the next consisted of age labels (e.g., toddler, preschool, child), and the last consisted of names of social-communication measures (e.g., Preschool Language Scales, Social Responsiveness Scale). The list of measures was generated by reviewing previously published reviews (Bolte & Diehl, 2013; McConachie et al., 2015), and as such, more recently developed measures were not included. We use the term “measure” throughout to describe both the scales and subscales that make up the measure. The scales and subscales of measures are often analyzed and discussed separately and so are also analyzed here as separate units. Searches of each database took place in August 2017, June 2018, and May 2020.

#### Inclusion Criteria

Inclusion criteria for studies were: (a) an experimental or quasi-experimental design, such as a randomized controlled trial or other longitudinal study (including observational studies), (b) enrollment of participants with a confirmed diagnosis of autism (including previous diagnoses of Asperger’s Syndrome under the Diagnostic and Statistical Manual of Mental Disorders-IV (DSM-IV). Children could be diagnosed with a clinical measure such as the Autism Diagnostic Interview-revised (ADI-R; Rutter, Couteur & Lord, 2003), the Autism Diagnostic Observation Schedule (ADOS; Lord et al., 2012), by a record review or by school personnel, (c) mean age of the participants between 24 months and 96 months of age (this age range was selected because 8 years old represents the end of the early intervention period in many systems), (d) a social-communication measure administered at two time points or more and separated by at least two weeks and (e) published between 1990 and 2020. Dissertations were included.

#### Study Selection

**Fig. 1:**
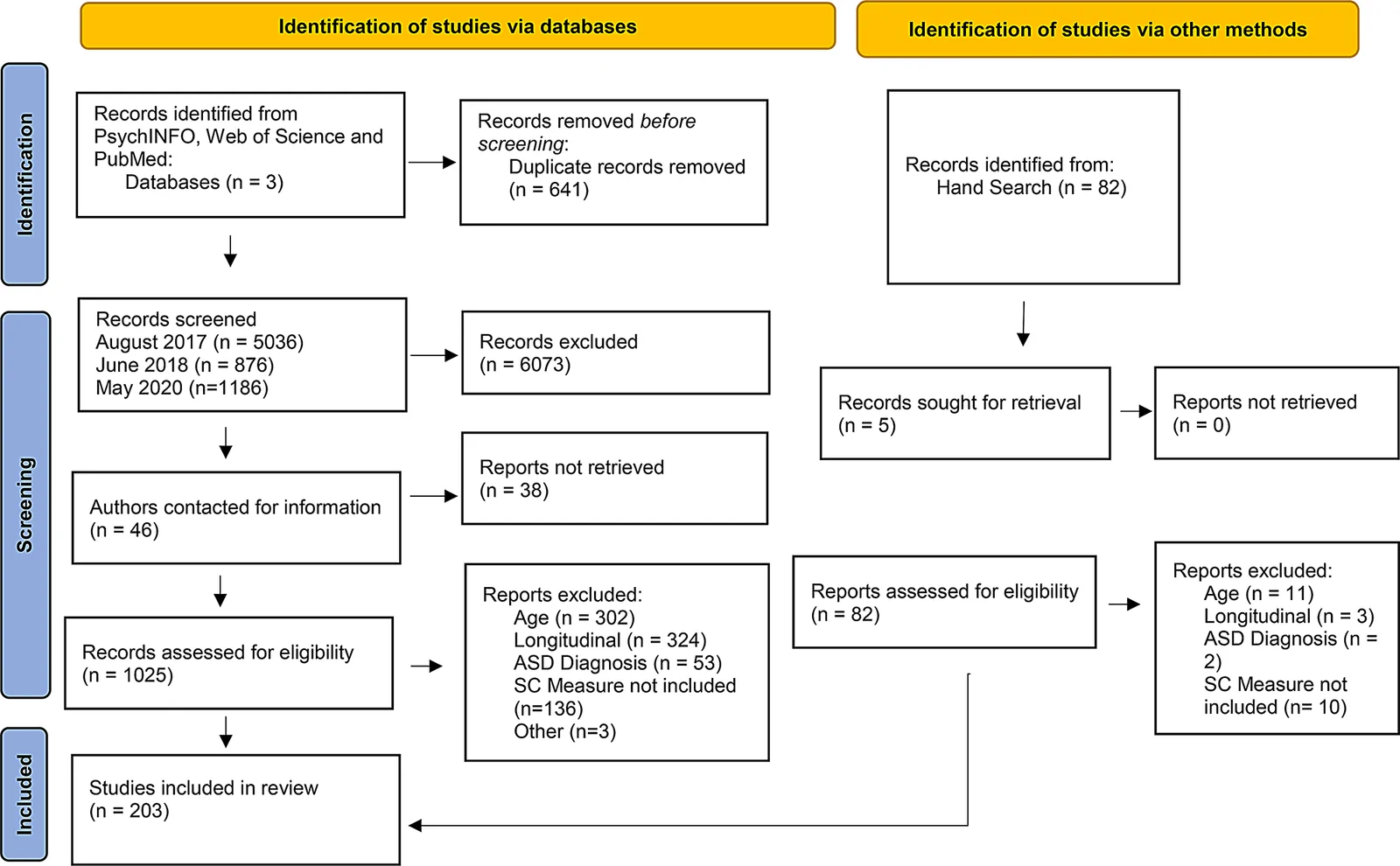
PRISMA Flow Diagram for Systematic Search Procedure. *Note. From*: Page MJ, McKenzie JE, Bossuyt PM, Boutron I, Hoffmann TC, Mulrow CD, et al. The PRISMA 2020 statement: an updated guideline for reporting systematic reviews. BMJ 2021;372:n71. doi: https://doi.org/10.1136/bmj.n71

The study selection process took place over three stages. First, title and abstracts were screened to identify clear violations of the inclusion criteria. Next, full texts were reviewed to confirm the studies met the full set of inclusion criteria. Lastly, a hand search was completed. Reference lists of the included studies, and the reference lists of other reviews and meta-analyses, were evaluated for additional studies to be included. After saturation was reached, the articles identified in the hand search were subjected to the inclusion criteria using the same procedures described above. A full description of the inclusion process is provided in Fig. 1. Any duplicate studies were removed at this point as well.

### Data Extraction

Dependent and independent variables were selected a-priori based on previous literature and extracted from each study. These included (a) length of the measurement period, (b) the way the outcome was scored, (c) chronological age, (d) intervention received and (e) study quality. A list of the variables and data that were extracted, along with definitions, are provided in Table 1. Study selection and data extraction was completed by the first author and second author, both advanced doctoral students in psychology.

**Table 1:** Extracted variables and operationalizations

Variable	Definition
Type of Study	Whether the study was a dissertation or peer reviewed article.
Name of Intervention	If applicable, the name of the intervention package or type of intervention that the child received (if any).
Sample Size	The total sample size of each of the groups.
Diagnostic Procedure	The method of diagnosis for the children enrolled in the study.
Sample Age	The mean age of the children in the sample.
Gender	The percentage of male subjects in the sample.
Length of Study	The total amount of time between each measurement period.
Type of Measure	Whether the reported scores are standard, raw, age-equivalent or developmental quotient scores.
Study Quality	Whether the study met the criteria for each of the 13 study quality questions. Summary rating based on the 13 questions.

#### Length of the Measurement Period (Length)

This represents the total time in months from baseline to follow-up or from one follow-up period to another. For example, from an intervention trial with a two-month intervention phase and a four-month follow-up, two lengths would be extracted --- the two-month period from entry to exit and the four-month period from exit to follow-up.

#### Type of Outcome

When appropriate, outcomes were classified based on whether they were raw scores, age-equivalent scores, developmental quotients, or standard scores.

#### Intervention Received

Due to extensive variability in the types of services received during the measurement periods, intervention data were categorized based on whether the sample was receiving treatment as usual (TAU), an identifiable behavioral intervention (Behavioral), or receiving medication as a part of a pharmacological trial, including open label trials (Medication). While the TAU group could have been receiving outside services during the measurement period, there was unreliable reporting of the amount and intensity of these services. Therefore, the variable should not be viewed as intervention versus no intervention. Rather, as: identifiable research-based intervention, community-based services, or the medication arm of a pharmacological trial versus non-identified community intervention. In the case where a study included two active treatments, both treatment arms were analyzed separately.

#### Study Quality

The Quality Assessment Tool for Observational Cohort and Cross-Sectional Studies was used to evaluate the methodological quality of the included studies (National Institutes for Health, 2019). This scale allows for both controlled and observational studies to be rated on the same scale. It has 14 items, but one item related to the participation rate in the sample was excluded as this information is rarely provided or available. This left 13 items that were rated as “Yes”, “No” or “NA” (CD/NA/NR; cannot determine, not applicable or non-reported). The information from the individual items is used to generate a holistic rating of study quality that categorizes studies as, “Poor”, “Fair” or “Good.” An example of a study that met the “Poor” rating was one that had greater than 20% attrition, assessors were not blind to timepoint, confounds were not adjusted for and the sample description was missing key information. An example of a study that met the “Fair” rating was one that had greater than 20% attrition and did not control for confounds but did have blinded assessors and a well-described sample. All variables from the 13 item tool were used to make the global rating.

### Reliability

At the study screening stage approximately 3% of the 7098 articles were reviewed together by the authors, which was approximately 20 articles each week while articles were being actively screened. Almost all discrepancies were in the direction of including articles that would not meet the inclusion criteria after further review, and so the decision was made to not increase the total percentage of articles that were screened twice. The authors discussed any discrepancies weekly at this stage to come to a consensus. At the study inclusion and extraction stage, approximately 10% of studies were double-coded by two independent coders. Inter-rater reliability was run using percent agreement and Kappa statistics.

### Analysis

The meta-analysis portion of the study followed the guidelines of Harrer et al. (2019a, b) and Borenstein et al. (2021). This procedure consists of five steps: (a) calculating the effect sizes for the study groups, (b) pooling the effect sizes, (c) identifying influential cases, (d) running subgroup and meta-regression analyses, and (e) assessing publication bias. Only measures with more than 12 effect sizes contributing to the pooled total and that represented the constructs of expressive and receptive language, social interactions, and social communication were included in these analyses. The conservative cut-off of 12 effect sizes was chosen to provide stable estimates in the meta-regression analyses. Previous studies have shown that at least 5 effect sizes are needed to generate informative pooled estimates (Valentine et al., 2010). Including measures with at least 12 effect sizes meant that there would be greater than 5 effect sizes per group in the subgroup analyses. The “Metafor” package (Viechtbauer, 2010) in R (version 4.0.2; R Core Team, 2020) was used to estimate the pooled effects. Evaluation of outliers and influential statistics and supplementary analyses was done using the “Dmetar” package (Harrer et al., 2019a, b). All data and code are available via GitHub by request.

### Primary Aim: Effect Size of Change Over Time

#### Effect Size Calculations

Hedges’g (Hedges & Olkin, 2014) was used to represent the standardized mean difference (*SMD*) score for each group calculated using unadjusted means. A group was defined as either a study, or in the case of intervention trials, each group was an arm within the trial. When there were more than two measurement time points within a group, separate standardized mean difference estimates were calculated for the difference between the first and second time points and the second and third time points.

#### Pooling the Effect Sizes

A random effects model using the Hartung-Knapp-Sidik-Jonkman estimator (Sidik & Jonkman, 2005) was used to estimate the pooled effect size and confidence intervals for the pooled effect sizes. This estimator provides a more conservative estimate than other methods (DerSimonian & Laird, 1986) and has been shown to be less biased in cases of high levels of heterogeneity between studies (Sidik & Jonkman, 2007). The standard errors for the pooled effects were estimated with Knapp-Hartung adjustments (Knapp & Hartung, 2003).

To address issues around independence, a third model was fit for each outcome to account for the multi-level structure of the data, and to determine whether “study” accounted for a significant portion of the variance in the pooled effect size estimate. This was necessary because some studies contributed multiple effect sizes to the pooled estimate. The structure of this model was as follows: Participant at level 1, Group at level 2, and Study at level 3. These models were fit using the restricted maximum-likelihood (REML; Viechtbauer, 2005) estimator. If level 3 did not explain a significant portion of the variance, and did not improve model fit, then the simple random effects model was used.

#### Outliers and Influential Cases

A group was considered an outlier if the 95% confidence interval for the effect size of an individual study did not cross the 95% confidence interval of the pooled estimate. Influential cases were identified using visual inspection and the leave-one-out method (Viechtbauer & Cheung, 2010a, b).

#### Publication Bias

Publication bias was assessed through a visual inspection of the funnel plots along with Egger’s test (Egger et al., 1997). The unit of analysis was the groups within studies, and not the comparison of change between the groups. Therefore, the central marker of the funnel plot was not set at “0” but at the average pooled effect size for the measure. A conservative re-estimation based on the Duval and Tweedie trim and fill procedure (Duval & Tweedie, 2000) was used only in cases of extreme or unexpected deviance based on substantive expectations.

### Secondary Aim: Predictors of Change

#### Meta-Regression Procedure

A multi-step process was used to evaluate the relationship between the continuous predictors (i.e., Length and chronological age), categorical predictors (i.e., study quality, intervention type, type of outcome), and the overall pooled effect size. Each predictor was interacted with the Length variable. If that interaction term was non-significant, then the interaction term was removed, and only simple main effects were included in the final model. The restricted maximum likelihood estimator was used to generate the effect size estimates for models that included interaction terms.

## Results

### Study Screening

Across the three databases, 7098 articles were culled for review and the hand search identified an additional 82 articles. 6073 studies were excluded at the abstract screening phase and full text review excluded an additional 820 articles. A total of 203 articles met full inclusion criteria and contributed data to the meta-analysis. A full list of included studies, including citations, is provided in Online Resource 1. Refer to Fig. 1 for a flow diagram of the screening process. Of the 203 included studies, 13 were dissertations and 190 were peer-reviewed articles. Descriptive statistics for the included sample are provided in Table 2.

**Table 2:** Descriptive information from included studies

	Descriptive Information: Continuous Variables		
	Mean (SD)	Median	Range
Sample Size	34.26 (29.20)	26	4–421
Chronological Age (months)	49.97 (10.58)	49	21- 99.6
Study Year	2013 (5.31)	2015	1990–2020
Length (months)	7.82 (8.94)	6	0.23–48

	Descriptive Information: Categorical Variables		
	n		
**Type of Article**			
Peer Reviewed	190		
Dissertation	13		
**Diagnostic Instrument**			
Record Review	61		
ADI-R Only	14		
ADOS Only	65		
Both ADI-R and ADOS	65		

Note. Length = Length of the Measurement Period, ADI-R = Autism Diagnostic Interview-Revised, ADOS = Autism Diagnostic Observation Schedule

Across studies, 119 unique social-communication measures were identified, and there were a total of 1232 effect sizes pooled across all measures. A full list of measures is included in Appendix B. Many measures were used in only one study (*n* = 55). Thirty-five measures were not included because they did not have a sufficient number of effect sizes. A list of measures for which effect sizes were pooled is provided in Table 3 along with the number of effect sizes and average Length.

**Table 3:** Pooled effect size across included studies

Measure Name	Number of Effect Sizes	Mean Length	SD Length	Overall Random Effect	Lower CI	Upper CI	Adjusted Random Effect	Adjusted Lower CI	Adjusted Upper CI	Test Retest
ADOS CSS*	47	16.79	13.48	-0.114	-0.2203	-0.0078	-0.1146	-0.2179	-0.0113	0.71-0.87^a^
ADOS-2 Social Affect/ ADOS-G Social*	38	10.68	8.37	-0.3243	-0.4441	-0.2044	-0.3772	-0.4725	-0.2819	0.78
ADOS-2 Communication and Language/ ADOS-G Communication*	20	7.21	4.95	-0.6155	-0.8565	-0.3746	-0.6624	-0.8995	-0.4254	0.73
ADOS-2 SI, ADOS-G Social*	18	6.57	4.8	-0.4904	-0.7404	-0.2404	-0.6262	-0.8381	-0.4142	0.82
ESCS IJA	42	5.77	4.29	0.1936	0.0866	0.3005	0.1936	0.0866	0.3005	0.79–0.91
MCDI Comprehension	33	7.55	5.012	0.4027	0.2842	0.5213	0.3721	0.258	0.4862	0.87
MCDI Expressive	49	6.72	4.68	0.4112	0.3102	0.5121	0.4153	0.3129	0.5177	0.95
Mullen Expressive	59	10.7	7.03	0.5608	0.4101	0.7115	0.4385	0.3264	0.5505	0.83-0.99^b^
Mullen Receptive	40	10.61	6.54	0.6494	0.463	0.8358	0.5183	0.3812	0.6554	0.83-0.99^b^
PLS Expressive	17	7.43	8.35	0.2875	0.1503	0.4247	0.2394	0.1464	0.3325	0.86–0.95
PLS Receptive	13	8.86	9.13	0.3748	0.1644	0.5822	0.2571	0.1329	0.3812	0.86–0.95
Reynell Expressive	29	8.11	4.56	0.552	0.3789	0.7251	0.5107	0.3543	0.6671	0.96
Reynell Receptive	30	8.04	4.49	0.4778	0.3415	0.6141	0.4319	0.3155	0.5483	0.91
SRS Total*	77	4.67	3.71	-0.2623	-0.3418	-0.1829	-0.2318	-0.2933	-0.1703	0.88
SRS Cognition*	36	4.71	3.17	-0.2055	-0.3259	-0.0851	-0.2321	-0.3244	-0.1398	-
SRS SA*	30	4.76	3.34	-0.1648	-0.2578	-0.0718	-0.1648	-0.2578	-0.0718	-
SRS SC*	38	5.58	4.24	-0.2523	-0.3214	-0.1831	-0.2523	-0.3214	-0.1831	-
SRS SM*	38	5.12	3.48	-0.3123	-0.4195	-0.205	-0.283	-0.3754	-0.1906	-
Vineland Communication	110	10.33	8.81	0.367	0.2769	0.4571	0.3445	0.2627	0.4263	0.76–0.92
Vineland Socialization	94	9.96	8.4	0.3581	0.2709	0.4453	0.3635	0.2771	0.45	0.76–0.92

Note. ADOS = Autism Diagnostic Observation Schedule, ESCS = Early Social Communication Scales, IJA = Initiations of Joint Attention, Length = Length of the Measurement Period, PLS = Preschool Language Scales, MCDI = Macarthur Communicative Development Inventories, SA = Social Awareness, SM = Social Motivation, SC = Social Communication, SI = Social Interaction, SRS = Social Responsiveness Scale

^a^ These scores represent the test-retest reliability across the 5 modules

^b^ These scores represent test-retest reliability across 3 age groups (24, 30–42, 48–66 months)

* A reduction in the scores on these measures over time represents less autism related behaviors

### Reliability

242 articles were double-coded during the screening phase, the percent agreement on whether to include an article was 83.4% and the Kappa was 0.52. During the inclusion phase, 181 articles were double coded, the percent agreement was 93.9%, and the Kappa was 0.71 based on the decision to include or not include a particular study. Seventeen studies that met full inclusion criteria were double-coded during the data extraction stage, and the percentage of individual decisions with exact agreement across extracted variables was 82.7%. When looking at the study quality ratings specifically, percent agreement was 81.9%.

### Outcomes

For each outcome, the univariate influence of Length on change over time was modeled first. Then, separate models with child age, study quality, study year, intervention type, and outcome scoring type were fit alongside Length. All models were fit for each outcome, and only statistically significant parameters are reported below. The third multi-level model that was fit to account for the “study” level lack of independence did not improve fit in any of the models and is therefore not reported below. Unless otherwise noted, positive slope parameters indicate improvements in behaviors.

#### Expressive Language

##### MacArthur-Bates Communicative Development Inventories (MCDI)- Expressive (Fenson, 2007)

The effect of Length was non-significant (*b* = 0.01, *SE* = 0.01, *p* =.27). However, there was an effect of study quality. Those studies rated as “Poor” had the largest effect sizes (*k* = 12, *SMD* = 0.69, 95% *CI* [0.45; 0.93]) compared to those rated as “Fair” (*k* = 22, *SMD* = 0.37, 95% *CI* [0.21; 0.53]) and “Good” (*k* = 14, *SMD* = 0.37, 95% *CI* [0.20; 0.55]).

The effect of chronological age was significant. For every additional month, there was an expected decrease of the *SMD* (indicating less expected improvement in language) of 0.02 units, (*b*=-0.02, *SE* = 0.004, *p* <.0001), however, the interaction between chronological age and Length was significant (*b*= -0.03, *SE* = 0.0009, *p* <.001). Younger children, on average saw more change in shorter study periods than older children, this effect was not present in longer studies.

##### Mullen Scales of Early Learning (MSEL)- Expressive (Mullen, 1995)

There was a significant effect of Length, where a 1 month increase in Length leads to an expected increase of approximately 0.02 effect size units (*b* = 0.02, *SE* = 0.01, *p* =.03).

The estimated pooled effect was larger for the TAU group than the behavioral intervention group, although the difference was non-significant (*k* = 28, *SMD* = 0.54, 95% *CI* [0.34; 0.74] and *k* = 26, *SMD* = 0.33, 95% *CI* [0.22; 0.44], *p* =.06). The difference between the behavioral intervention and TAU groups was further accounted for when Length was included in the model (*p* =.49). There was also a main effect of type of measure (*p* =.02) where age equivalent scores had the largest effects (*k* = 23, *SMD* = 0.58, 95% *CI* [0.40; 0.76]), compared to standard scores (*k* = 15, *SMD* = 0.30, 95% *CI* [0.10; 0.50]) and developmental quotients (*k* = 16, *SMD* = 0.30, 95% *CI* [0.16; 0.44]).

##### Preschool Language Scales (PLS)- Expressive (Zimmerman, 2011)

One case was removed because the length of the measurement period was 36 months longer than the next closest study. With this case removed, there was a significant effect of Length on the pooled effect size, where the effect size was expected to increase by 0.04 *SMD* units for every additional month (*b* = 0.04, *SE* = 0.01, *p* <.0001).

The Eggers’ Test was significant (*p* =.01). However, the funnel plot showed that the skew was driven by studies with smaller effects rather than larger effects, therefore it is not likely that this skew can be attributed to publication bias.

##### Reynell Developmental Language Scales (Reynell)- Expressive (Reynell, 1990)

There was one case that was removed because the length of the measurement period was 11 months longer than the next closest study. With this case removed, there was a significant effect of Length (*b*=-0.05, *SE* = 0.02, *p* =.01). The effect size was expected to decrease by 0.05 *SMD* units. This indicates less expected growth in language as measured by this measure in longer studies.

##### Autism Diagnostic Observation Schedule (ADOS-2)- Language and Communication (Lord Et Al., 2012) and ADOS-G Communication (Lord Et Al., 2000)

In the simple meta-regression models the effect of Length was significant (*b*=-0.06, *SE* = 0.02, *p* =.01); for every additional month there was an expected decrease in the average effect size of approximately 0.06 units (the average effect size gets more negative). Decreases on this measure and a more negative effect size indicate fewer autism related behaviors. When chronological age and Length were included together in the model, age was significantly related to the *SMD* (*b* = 0.02, *SE* = 0.01, *p* =.05), which means that less change in autism related behaviors is expected in older children. Length was no longer significant in the model (*b*=-0.003, *SE* = 0.03, *p* =.92) which means that expected change is better explained by children’s age than the amount of time between measurement periods.

The effect of intervention group approached significance (*p* =.06). Those receiving a medication-based treatment (*k* = 4, *SMD*=-0.30, 95% *CI* [-0.95; 0.35]) had the lowest expected change compared to the TAU (*k* = 5, *SMD*= -0.88, 95% *CI* [-1.25; -0.50]) and behavioral intervention groups (*k* = 10, *SMD*= -0.70, 95% *CI* [-1.09; -0.31]).

#### Social Interaction

##### ADOS-2 Reciprocal Social Interaction (Lord Et Al., 2012) and ADOS-G Social (Lord Et Al., 2000)

There was a significant effect of Length (*b*=-0.04, *SE* = 0.02, *p* =.04). For every additional month, the effect size is expected to decrease by approximately 0.04 units. Again, decreases on this measure and a more negative effect size indicate fewer autism related behaviors. There was also a significant effect of age (*b* = 0.02, *SE* = 0.004, *p* =.002). For every month increase in age, the effect size for ADOS-2 Reciprocal Social Interaction scores is expected to increase by approximately 0.02 units. This means there is expected to be less change in autism related behaviors in older individuals (the effect size is less negative and closer to 0).

Lastly, there was a significant interaction between type of intervention received and Length where those receiving medication made more change over time than those receiving behavioral interventions (*b*=-0.58, *SE* = 0.16, *p* =.005). However, there were only three effect sizes in the medication group, so this contrast should be treated with caution.

##### Social Responsiveness Scale (SRS)- Social Awareness (Constantino & Gruber, 2012)

The effect of Length was non-significant (*b* = 0.002, *SE* = 0.01, *p* =.89). There was an effect of type of intervention (*p* =.02). Those receiving TAU had the smallest *SMD* (*k* = 11, *SMD*= -0.02, 95% *CI* [-0.13; 0.10] compared to behavioral interventions (*k* = 11, *SMD*= -0.26, 95% *CI* [-0.43; -0.10] and medications (*k* = 8, *SMD*= -0.18, 95% *CI* [-0.40; 0.04]. Negative *SMD* for all SRS related outcomes indicate fewer social challenges or social differences.

##### SRS- Social Motivation (Constantino & Gruber, 2012)

The effect of Length was non-significant (*b*=-0.01, *SE* = 0.14, *p* =.68), however, there was a significant interaction between Length and study quality where those studies rated as “Poor” saw less change than studies rated as “Good” (*b* = 0.15, *SE* = 0.06, *p* =.02). The difference between “Poor” and “Fair” studies verged on significance in the same direction (*b* = 0.10, *SE* = 0.06, *p* =.09). There was also a significant interaction between Length and type of intervention received. Those receiving medication saw greater reductions in scores as length increased compared to those receiving behavioral interventions (*b*=-0.07, *SE* = 0.03, *p* =.04). This interaction was no longer significant when controlling for study quality (*p* =.59).

The Eggers’ Test was significant (*p* =.01). On inspection of the plot, the skew was driven by two effect sizes that were extremely negative with no comparably large positive effects.

##### SRS- Cognition (Constantino & Gruber, 2012)

The effect of Length was not significant (*b*=-0.004, *SE* = 0.15, *p* =.80). However, there was a significant interaction between Length and study quality; those studies rated as “Poor” had a weaker relationship between Length and effect size than both studies rated as “Fair” (*b* = 0.13, *SE* = 0.05, *p* =.02) and studies rated as “Good” (*b* = 0.19, *SE* = 0.06, *p* =.002).

The interaction between type of intervention received and Length was significant; those in the behavioral intervention group saw larger effect sizes as length increased (*b*=-0.13, *SE* = 0.06, *p* =.03).

##### Vineland Adaptive Behavior Scales (VABS)- Socialization (Sparrow et al., 2005, 2016)

The effect of Length was not significant (*b*=-0.01, *SE* = 0.005, *p* =.26). The subgroup analysis comparing the types of intervention received was significant (*p* =.04). Those in the behavioral intervention group (*k* = 45, *SMD* 0.48, 95% *CI* [0.36; 0.60] had the largest effects, compared to both the TAU group (*k* = 33, *SMD* = 0.26, 95% *CI* [0.11; 0.42]) and the medication group (*k* = 15, *SMD* 0.27, 95% *CI* [0.07; 0.47]). There was a trend toward significance in the interaction between type of treatment received and Length where those receiving behavioral interventions saw more change as the length increased compared to those receiving TAU (*b*=-0.02, *SE* = 0.01, *p* =.07).

The effect of type of measure approached significance; the effects sizes derived from standard scores had the smallest effect (*k* = 69, *SMD* = 0.31, 95% *CI* [0.22; 0.41]) compared to both age equivalent scores (*k* = 13, 0.62 [0.33; 0.91]) and raw scores (*k* = 11, *SMD* = 0.42, 95% *CI* [0.17; 0.67]). However, there was a significant interaction between type of measure and Length where raw scores increased more as Length increased compared to age equivalent scores (*b* = 0.05, *SE* = 0.03, *p* =.04).

#### Social Communication

##### ADOS-2 Social Affect (Lord et al., 2012) and ADOS-G Social Communication (Lord et al., 2000)

The effect of Length was non-significant (*b*=-0.07, *SE* = 0.01, *p* =.24). This indicates that larger effect sizes are not expected as the length of the measurement period increases. No significant results emerged from the subgroup or meta-regression models, however, there were two studies with extremely long measurement lengths (40 months) and so the subgroup and meta-regression models were re-run without these studies. With these cases removed, there was a main effect of type of intervention received. The medication group had a larger expected effect size compared to the behavioral intervention group that approached significance (*b*=-0.47, *SE* = 0.25, *p* =.07) and there was a significant main effect of Length (*b*=-0.02, se = 0.01, *p* =.01) when controlling for type of intervention received. For every additional month, the effect size is expected to decrease by approximately 0.02 *SMD.* This means there is a larger expected decrease in autism related behaviors in longer studies. In the simple meta-regression model, Length was non-significant (*b* = 0.02, *SE* = 0.01, *p* =.10).

Egger’s test was significant (*p* <.001). However, on inspection, the pattern observed in the funnel plot did not seem to be driven by publication bias, but rather by the fact that there were no studies that reported extreme increases in autism related behaviors. It is therefore unlikely that publication bias is present in the reporting of this measure.

##### ADOS- Calibrated Severity Scores (CSS; Gotham et al., 2009)

The effect of Length approached significance (*b* = 0.0068, se = 0.0036, *p* =.07). For every additional month, there was an expected increase in the effect size of approximately 0.01 units. A positive effect here means that there is less expected change over time because the average effect size for ADOS CSS scores is negative. However, the interaction between Length and type of intervention received was significant. Those receiving a behavioral intervention experienced more change as Length increased compared to those receiving TAU (*b* = 0.02, *SE* = 0.0071, *p* =.04). It is important to note that ADOS CSS scores are inclusive of items measuring restricted and repetitive behaviors as well.

##### Early Social Communication Scales (ESCS)- Initiations of Joint Attention (Seibert et al., 1982)

The effect of Length approached significance (*b* = 0.02, *SE* = 0.12, *p* =.08). For every additional month, one would expect the effect size for ESCS IJA to increase by 0.02 units. No other parameters were significant in the models.

##### SRS- Social Communication (Constantino & Gruber, 2012)

The effect of Length was non-significant (*b* = 0.001, *SE* = 0.01, *p* =.89). However, the interaction between Length and type of intervention received was significant; the medication group saw a greater reduction in scores as Length increased (*b*=-0.02, *SE* = 0.012, *p* =.005).

##### VABS- Communication (Sparrow et al., 2005, 2016)

The effect of Length was non-significant (*b* = 0.001, *SE* = 0.004, *p* =.901), and the effect of types of intervention received approached significance (*p* =.07). Those receiving a behavioral intervention (*k* = 56, *SMD* = 0.44, 95% *CI* [0.35; 0.53] had the largest effect, compared to the TAU (*k* = 41, *SMD* = 0.25, 95% *CI* [0.10; 0.39] and Medication groups (*k* = 12, *SMD* = 0.27, 95% *CI* [-0.12; 0.66]. However, there was a significant interaction between type of intervention and Length, where those in the medication group saw more change as Length increased compared to the behavioral group (*b* = 0.15, *SE* = 0.05, *p* =.001). The contrast between the behavioral and TAU groups was non-significant (*b*=-0.003, *SE* = 0.01, *p* =.77).

There was also a significant effect of type of measure (*p* <.001). The effects sizes derived from standard scores had the smallest effect (*k* = 88, *SMD* = 0.29, 95% *CI* [0.20; 0.39]) compared to both the age equivalent (*k* = 14, *SMD* 0.55, 95% *CI* [0.42; 0.68]) and raw scores (*k* = 5, *SMD* = 0.71, 95% *CI* [0.24; 1.18]).

## Discussion

The need for valid and reliable measures that accurately measure progress across complex behavioral phenomena, and to understand the influence of demographic factors on change in outcomes over time has been well-documented (Charman et al., 2003; Grzadzinski et al., 2020; Kasari, 2002; Lord et al., 2005). The rapid proliferation of interventions for young autistic children, including the high quality interventions that many children receive in the community that were once exclusive to research (Rogers et al., 2019), has also led to the rapid proliferation of measures to evaluate those interventions. There are 131 to 289 different outcome measures used to assess young autistic children, depending on the constructs included (Bolte & Diehl, 2013; McConachie et al., 2015). Many of those measures, such as the ADOS and SRS, were not designed to measure change over time. In questionnaires the instructions for windows of time that behaviors should be reported on are rarely confirmed with those completing the measure. As an example, reporting on the previous 6-months for the SRS includes time the child has spent both in and out of intervention in many cases. These data point to the need for thoughtful consideration of the choice of outcome measures to ensure they are appropriate to address a study’s aims. Collecting data on children’s progress over time, both in research and community care is critically important. In this study, we evaluated the sensitivity to change and expected change over time of commonly used measures of social communication. This information can be used to address practical questions such as whether it is appropriate to pool together different measures of similar constructs, and whether the way a measure is scored influences its sensitivity to change.

### Sensitivity to Change

Using length of the measurement period as a predictor of effect size provided a metric to examine sensitivity to developmental change over time. There were only five measures whose effect sizes were related to the length of the measurement period: ADOS-2 Communication and Language/ADOS-G- Communication, ADOS-2 Reciprocal Social Interaction/ADOS-G Social, Mullen Expressive Language, PLS Expressive Language and Reynell Expressive Language. The magnitude of these effects varied from negligible, an increase per month of 0.006 *SMD* units, to small, an increase of 0.06 *SMD* for each additional month. In other words, in a three-month period, the expected change using these measures would range from an effect size of 0.018 to 0.18, in those few measures where there was a relationship between length of the study and change over time.

Effect size estimates alone are not indicators of the importance of the change being made. However, these results do indicate that depending on the measure selected, one should not expect substantial differences in the overall effect size in shorter versus longer studies. There is a relatively weak relationship between the amount of change and the length of measurement period. This may be an indication that most change in interventions happens early in the treatment course (Smith et al., 2015; Virués-Ortega et al., 2013). It may also be that there was a difference in the dose of intervention in shorter and longer studies that accounted for this finding. Although, this is not a likely explanation given the mixed evidence that variations in dose are related to positive outcomes in early interventions (Estes et al., 2015; Rogers et al., 2021; Virués-Ortega, 2010). However, there is not enough information to assess whether the number of hours or frequency of intervention sessions differed across studies, so this remains a direction for future research. Interestingly, for Reynell- Expressive Language scores less change was expected as time increased. The Reynell uses standard scores, which could explain this effect. Children could be failing to keep up with norms rather than it being an indication that they were not making improvements over time.

Overall, these data indicate that most of the commonly used social-communication measures may not be sensitive to incremental change over time or simply that they are measuring stable developmental constructs like the amount of autism related behaviors (Gotham et al., 2012; Thurm et al., 2015). Those measures that were found to be sensitive to change over time were largely measuring language. In young children progress in language over time is expected in many children (Tager-Flusberg, 2018). While language delays commonly co-occur alongside autism they are not a core diagnostic feature. These results may indicate that core diagnostic features, such as social communication, may be more stable over time in young children, rather than showing a lack of sensitivity of the measures themselves. Increased attention and resources are needed to develop social-communication measures that are specifically designed to capture subtle change over time.

### Pooling of Measures Within Constructs

Overall, there was a substantial amount of variability in the expected change over time of specific measures within the broader constructs of expressive language, social interactions, and social communication. For example, the average effect sizes for the VABS Communication domain were 0.44 and 0.25 for those in behavioral intervention and TAU respectively, 0.22 and 0.27 for the PLS Expressive domain and 0.52 and in 0.43 for the Reynell Expressive domain. Not only do these measures of a similar construct have a different magnitude of expected change over time, but they may also be differentially sensitive to change within treatment trials. Measuring communication with PLS Expressive Language domain results in an expected change about half of as large as the Reynell Expressive Language domain. The practice of combining different measures of similar constructs is common in recent meta-analyses evaluating the efficacy of early interventions for autism (Nahmias et al., 2019; Sandbank et al., 2020). These results suggest that there should be additional empirical evidence to confirm that measures perform similarly across time and across children with different clinic characteristics before they are analyzed together.

### Treatment

Overall, there were very few measures that differentiated between the TAU and behavioral intervention groups. The MSEL- Expressive domain was the only non-parent report or interview-based measure for which there was a significant difference between TAU and behavioral intervention groups. Some domains of parent report measures or interviews such as the SRS and the VABS, also showed larger effects on average within the behavioral intervention groups. These data support the claim that most social-communication measures currently available to early intervention researchers lack the sensitivity to detect differential change as a result of receiving treatment (Anagnostou et al., 2015). Alternatively, it could be that the measures are not appropriately tailored to the available treatments being provided (i.e., they are measuring the wrong constructs based on what was targeted in the intervention). Without valid measures, our understanding of the evidence base for early intereventions remains stymied. Continued efforts to develop measures specifically designed to track change over time, such as the Brief Observation of Social Communication Change (BOSCC; Grzadzinski et al., 2016; Kim et al., 2019), therefore represent an important step in the early intervention literature for young autisitc children.

### Types of Measures

These data corroborate previous investigations showing that standard scores are generally less sensitive to change over time than raw and age-equivalent scores (Carter et al., 1998; Williams et al., 2006). Across both the VABS Communication and Socialization domains, and the MSEL Expressive domain, the smallest effects were seen across standard scores, on average about half of the expected change over time as age equivalent scores. There was also some indication that raw, and age equivalent scores change more as the length of the measurement period increased (a potential indication of sensitivity to change). The use of standard scores may attenuate observed change when used as an outcome measure. It is therefore advisable to report both standard and age-equivalent scores when possible.

### Age

Taken together, there was little indication that age influenced the magnitude of change over time or the sensitivity to change over time of social-communication measures. There were two exceptions. First, across the ADOS Reciprocal Social Interaction and Language and Communication domains, there is less expected change in older children. This is likely best explained by previously noted limitations in the use of ADOS raw scores as a metric of change in autism related behaviors over time (Gotham et al., 2007), which was one of the reasons for the development of calibrated severity scores (Gotham et al., 2009).

In shorter studies, the MCDI Expressive domain showed more change over time in younger compared to older children. Based on the inclusion criteria, the youngest children in this meta-analysis were approximately 2–3 years old. Considerably more growth in language abilities is expected in children younger than age 6, both in typically developing children, and autistic children (Anderson et al., 2007; Pickles et al., 2014). It is therefore unsurprising that growth would be observed in a shorter time period in younger versus older children.

### Limitations and Future Directions

One limitation of these data, as is true of any meta-analysis, is the variable methodological quality of the studies from which the data were extracted. While study quality was included as a covariate, and small sample studies are weighted differently in the pooled estimates, concern for undue influence based on study quality remains. There may also be variability in the effectiveness of the interventions used within the studies. If higher-quality interventions tended to use specific outcomes, this could bias the results. Second, complete and adequate data were not accessible from a number of studies (e.g., means and standard deviations for the outcomes of interest), and so they could not be included in this meta-analysis. It is possible that the observed relationships or patterns of findings could shift with the inclusion of these studies. Other missing data issues, such as not having adequate information to determine the developmental appropriateness of the selection of measures within each study also could bias our conclusions. Third, while the analyses of effects within measures (rather than pooled across different measures) is a strength of the study, it did limit the power to analyze measures that were less frequently present in the included studies. As a result, less definitive conclusions could be drawn about some measures. Fourth, meta-regression and sub-group analyses using the average of participant-level characteristics (i.e., average age of sample) is a crude metric. This is especially true if there is variability within the sample within that characteristic (i.e., a wide age-range). The results of these analyses should be considered exploratory, and used as a guide for participant-level analyses in the future. Lastly, no corrections for multiple comparisons were applied given the exploratory nature of these analyses. This increases the risk for false positive findings and replication of these results will be important.

These analyses should serve as an impetus for further detailed psychometric evaluations of participant-level data. One framework for such evaluations in health outcomes research is through the Item Response Theory framework (IRT; Edelen & Reeve, 2007). Recent studies have found success in refining or evaluating commonly used measures including the SRS (Sturm et al., 2017), the SCQ (Wei et al., 2015), ADOS (Kuhfeld & Sturm, 2017) and SSIS (Anthony et al., 2016), through this framework. It will be important to address issues concerning item difficulty. It is assumed that progress at all points of measures is equally difficult (e.g., making progress from item 5 to 8 on the Mullen Expressive Language domain is equally as difficult as making progress from item 15 to 18). This assumption remains largely untested, and may prove to be an important direction for future research. Further rigorous psychometric work is needed to answer this, and other clinically important questions.

## Conclusions

One of the unique aspects of this manuscript is the focus on specific measures as the unit of analysis, rather than pooling effect sizes across measures, an initial step toward the goal of increasing clarity and providing an explicit rationale for the selection of specific measures (Grzadzinski et al., 2020). The sheer number of measures available to assess social-communication outcomes in autistic children can be overwhelming. These data point to a siloed approach to the choice of appropriate outcome measures, with research groups rarely providing an explicit rationale for their selection of measures. A number of measures including the MSEL Expressive Language domain, the VABS, SRS, MCDI and ESCS were used fairly frequently. Although the effect sizes were small across the most commonly used measures, the MSEL Expressive Language domain and the VABS Socialization and Communication domain scores seemed to differentiate between behavioral and TAU groups well. The expected change for Mullen domain scores was large. This is particularly true when using age equivalent, rather than standard scores.

This study extends on previous reviews (Bolte & Diehl, 2013; McConachie et al., 2015) to better understand the breadth of available social-communication outcomes, and to specifically evaluate and provide a descriptive overview of two important psychometric characteristics of those measures; their expected change and sensitivity to change over time.

## Appendix A

### Search Terms and Strategy

The syntax used was modified for each individual database. The search syntax below represents a title and abstract search for the PsychINFO Database. First, broken up for clarity and then provided as it was entered.

### Syntax Broken up by Category

“Pervasive development* disorder*” OR Autis* OR PDD OR PDD-NOS OR Asperg* OR asd.

AND.

(Child* OR infan* OR kindergarten* OR pediatric OR toddler OR pre-school* OR preschool* OR “primary school*” OR “elementary school*”)

AND.

(“free play” OR “parent child interaction” OR “caregiver child interaction” OR “caregiver play interaction” OR PCX OR CCX OR “mother child interaction” OR “behavioral rating” OR “MacArthur Communicative Development Inventory” OR MCDI OR “Griffiths Scale of Infant Development” OR VABS OR “Vineland Adaptive Behavior Scale” OR “preschool language scales” OR PLS OR “Communication symbolic behavior scales” OR CSBS OR “Mullen Scales of Early Learning” OR M*SE*L OR “Leiter International Performance Scale” OR Leiter OR “Early intervention developmental profile” OR EIDP OR “Preschool Developmental Learning Accomplishments Profile” OR “Comprehensive Assessment of Spoken Language” OR CASL OR GMDS OR “Griffiths Mental Development Scales” OR BPVS OR “British Picture Vocabulary Scale” OR SICD OR “Sequenced Inventory of Communicative Development” OR DANVA OR “Diagnostic Analysis of Non-Verbal Accuracy” OR WASI OR “Wechsler Abbreviated Scale of Intelligence” OR SSRS OR “Social Skills Rating System” OR JAMES OR “Joint Attention Measure from ESCS” OR “Early Social Communication Scales” OR ESCS OR “Natural Language Sample” OR “structured play assessment” OR SPA OR “Social Communication Questionnaire” OR SCQ OR “Social responsiveness scale” OR “Imitation Battery” OR “Imitation disorders evaluation scale” OR “pre-verbal communication schedule” OR “social communication behavior codes” OR “Clinical Evaluation of Language Fundamentals” OR CELF OR “Expressive one-word picture vocabulary test” OR EOWPVT OR “receptive one-word picture vocabulary test” OR ROWPVT OR “Illinois Test of Psycholinguistic Abilities” OR “Peabody picture vocabulary test” OR PPVT OR “Battelle Developmental Inventory” OR “Bayley Scales of Infant Development” OR “Stanford-Binet Intelligence Scale” OR “autism diagnostic observation schedule” OR ADOS)

### Combined Search Syntax

TS=(“Pervasive development* disorder*” OR Autis* OR PDD OR PDD-NOS OR Asperg* OR asd) “AND” (Child* OR infan* OR kindergarten* OR pediatric OR toddler OR pre-school* OR preschool* OR “primary school*” OR “elementary school*”) “AND” (“free play” OR “parent child interaction” OR “caregiver child interaction” OR “caregiver play interaction” OR PCX OR CCX OR “mother child interaction” OR “behavioral rating” OR “MacArthur Communicative Development Inventory” OR MCDI OR “Griffiths Scale of Infant Development” OR VABS OR “Vineland Adaptive Behavior Scale” OR “preschool language scales” OR PLS OR “Communication symbolic behavior scales” OR CSBS OR “Mullen Scales of Early Learning” OR M*SE*L OR “Leiter International Performance Scale” OR Leiter OR “Early intervention developmental profile” OR EIDP OR “Preschool Developmental Learning Accomplishments Profile” OR “Comprehensive Assessment of Spoken Language” OR CASL OR GMDS OR “Griffiths Mental Development Scales” OR BPVS OR “British Picture Vocabulary Scale” OR SICD OR “Sequenced Inventory of Communicative Development” OR DANVA OR “Diagnostic Analysis of Non-Verbal Accuracy” OR WASI OR “Wechsler Abbreviated Scale of Intelligence” OR SSRS OR “Social Skills Rating System” OR JAMES OR “Joint Attention Measure from ESCS” OR “Early Social Communication Scales” OR ESCS OR “Natural Language Sample” OR “structured play assessment” OR SPA OR “Social Communication Questionnaire” OR SCQ OR “Social responsiveness scale” OR “Imitation Battery” OR “Imitation disorders evaluation scale” OR “pre-verbal communication schedule” OR “social communication behavior codes” OR “Clinical Evaluation of Language Fundamentals” OR CELF OR “Expressive one-word picture vocabulary test” OR EOWPVT OR “receptive one-word picture vocabulary test” OR ROWPVT OR “Illinois Test of Psycholinguistic Abilities” OR “Peabody picture vocabulary test” OR PPVT OR “Battelle Developmental Inventory” OR “Bayley Scales of Infant Development” OR “Stanford-Binet Intelligence Scale” OR “autism diagnostic observation schedule” OR ADOS).

## Appendix B

**Table B1:** Names of extracted social communication measures

ABAS-Social Subscale (1)	Bayley- Communication (1)	ESCS IJA (17)	MCDI Comprehension (16)	PEP-*R* Verbal Cognitive (2)	SSRS-Pos Social Behaviors (1)
ABBLS-R (1)	Bayley-Social Emotional (1)	ESCS RJA (7)	MCDI Expressive (23)	Reynell Total (2)	SSRS-Neg Social Behaviors (1)
ABC Total (1)	BOSCC Social Affect (4)	ESCS Requesting (8)	MCDI Gestures (3)	Reynell Expressive (10)	Symbolic Play Test (2)
ABC Social Withdrawal (1)	BOSCC Total (7)	Expressive Vocabulary Test (1)	MCDI MLU (2)	Reynell Receptive (11)	TOLD-3 Language (1)
ABC-Inappropriate Language (1)	CARS (15)	FEAS (3)	PLS Receptive (7)	ROWPVT (1)	TOPP-Pretend Play (1)
AEPS-SC (1)	CCC- Communication (1)	FEAS Developmental Questionnaire (1)	PLS Total (5)	SIB- Social Interaction (1)	Test of Playfulness (1)
AEPS-Social (1)	CCC Social Interaction (1)	GARS (2)	PIA Total (2)	SIB- Expressive (1)	VB-MAPP (1)
ADI-R-Communication (1)	CELF-4 (1)	GMDS Language (4)	PIA- Understanding (2)	SIB- Receptive (1)	VABS- Communication (57)
ADI-R-Social Interaction (1)	CDI (1)	GMDS Social (4)	PIA- Social Reciprocity (2)	STLP (1)	VABS- Expressive (6)
ADOS CSS (26)	CBRS Initiations (2)	GFTA (1)	PIA- Non-Verbal Communication (2)	SC Checklist- Engagement (1)	VABS- Receptive (5)
ADOS Social Affect/Social Communication (18)	CBRS Affect (1)	HKBABS Communication (1)	PDD-Behavioral Inventory (1)	SC Checklist- Language (1)	VABS- Socialization (47)
ADOS Language and Communication (10)	CBRS Interest (1)	HKBABS- SC (1)	PKBS- SC (1)	SC Checklist- Play (1)	
ADOS Play Items (2)	CBRS Joint Attention (2)	Joy and Fun Questionnaire (1)	PPVT-Receptive (9)	SCQ (5)	
ADOS Social Interaction/Social (9)	CSBS- SC (3)	PEP-R Overall Communication (1)	PPVT-Word Count (9)	SPA (10)	
ADOS Total (13)	CSBS Speech (2)	PJAM Finding Faces (1)	Merrill Palmer- R (1)	SPACE (2)	
ASQ Social-Emotional (1)	CSBS-Symbolic (1)	PJAM IJA (2)	Mullen Expressive Language (28)	SRS Total (35)	
ASQ Communication (1)	CSBS (3)	PJAM RJA (2)	Mullen Receptive Language (22)	SRS Cognition (15)	
Autism Symptom Rating Scale (1)	Developmental Profile 3 Communication (1)	PJAM Turn Taking (2)	Mullen Verbal Combined (4)	SRS-Social Awareness (14)	
ATEC-Language (2)	Developmental Profile 3 Social (1)	PLS Expressive (9)	PEP-R Expressive (2)	SRS-SC (17)	
ATEC-Social (2)	EOWPVT (4)	KTEA Oral Language (1)	PEP-R Receptive (2)	SRS Social Motivation (16)	
BASC-Social Skills (2)	ESCS Gestures (2)	KTEA Comprehension (1)		SSIS (3)	
BASC-Functional Communication (2)					

Note. The numbers in parentheses represent the number of unique studies in which a measure is represented. These measures were all identified in the search, but not all were included in the meta-analysis due to having too few effect sizes. ABAS = Adaptive Behavior Assessment System, ABBLS = The Assessment of Basic Language and learning Skills, ABC = Aberrant Behavior Checklist, AEPS = Assessment, Evaluation and Programming System, ADI = Autism Diagnostic Interview, ADOS = Autism Diagnostic Observation Schedule, ASQ = Ages and Stages Questionnaire, ATEC = Autism Treatment Evaluation Checklist, BASC = Behavior Assessment System for Children, BOSCC = Brief Observation of Social Communication Change, CARS = Childhood Autism Rating Scales, CCC = Children’s Communication Checklist, CBRS = Conner’s Comprehensive Behavior Rating Scales, CDI = Communicative Developmental Inventory, CSBS = Communication and Symbolic Behavior Scale, EOWPVT = Early One Word Picture Vocabulary Test, ESCS = Early Social Communication Scales, FEAS = Functional Emotional Assessment Scale, GARS = Gilliam Autism Rating Scale, GMDS = Griffiths Mental Development Scale, GFTA = Goldman-Fristoe Test of Articulation, HKBABS = Hong Kong Based Adaptive Behavior Scale, PEP-R = Psychoeducational Profile-Revised, PJAM = Precursors of Joint Attention Measure, PLS = Preschool Language Scales, KTEA = Kaufman Test of Educational Achievement, MCDI = Macarthur Communicative Development Inventories, PIA = Parent Interview for Autism, PDD = Pervasive-Developmental Disorder, PKBS = Preschool and Kindergarten Behavior Scales, PPVT = Peabody Picture Vocabulary Test, ROWPVT = Receptive One Word Picture Vocabulary Test, SC = Social Communication, SCQ = Social Communication Questionnaire, SIB = Scales of Independent Behavior, SPA = Structured Play Assessment, SRS = Social Responsiveness Scale, SSIS = Social Skills Improvement System, SSRS = Social Skills Rating System, STLP = Schlichting Test of Language Production, TOLD = Test of Language Development, TOPP = Test of Pretend Play, VB-MAPP = Verbal Behavior Milestones and Placement Program, VABS = Vineland Adaptive Behavior Scales

## Data Availability

All data and code are available by request through GitHub.
